# Supplementary material for: SEESAW: detecting isoform-level allelic imbalance accounting for inferential uncertainty
Source: Genome Biol. 2023 Jul 12;24:165. doi: 10.1186/s13059-023-03003-x (PMC10337143; doi:10.1186/s13059-023-03003-x)
Supplement: Supplementary file 1 — Additional file 1: Supplementary figures and tables. Additional file 1 includes 14 figures and 2 tables. The supplementary figures describe the pipeline of SEESAW, details on both simulated and osteoblast datasets, as well as benchmark results with existing methods. The tables display bootstrap coverage and the list of genes with discordant AI in the mouse F1 time course. [file 13059_2023_3003_MOESM1_ESM.pdf]

Additional file 1

Supplementary Figures and Tables

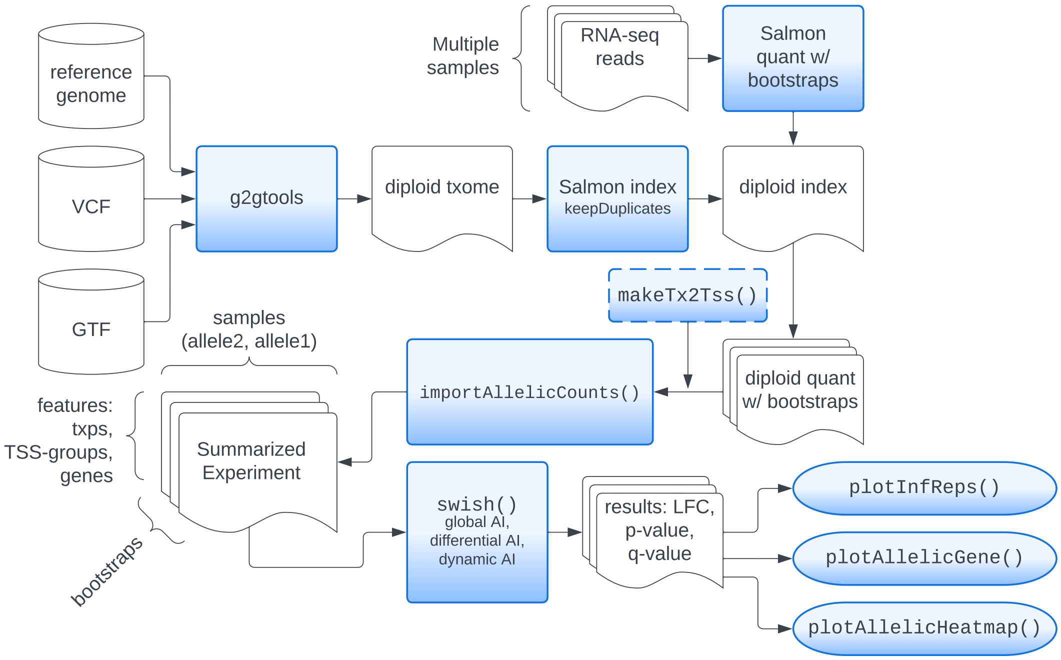

Figure S1: *SEESAW* pipeline for generating allelic expression estimates and performing statistical testing for allelic imbalance. After quantification with *Salmon*, the import and testing steps of *SEESAW* are available within the *fishpond* package on Bioconductor. *SEESAW* is accompanied with a detailed software vignette for performing allelic analysis.

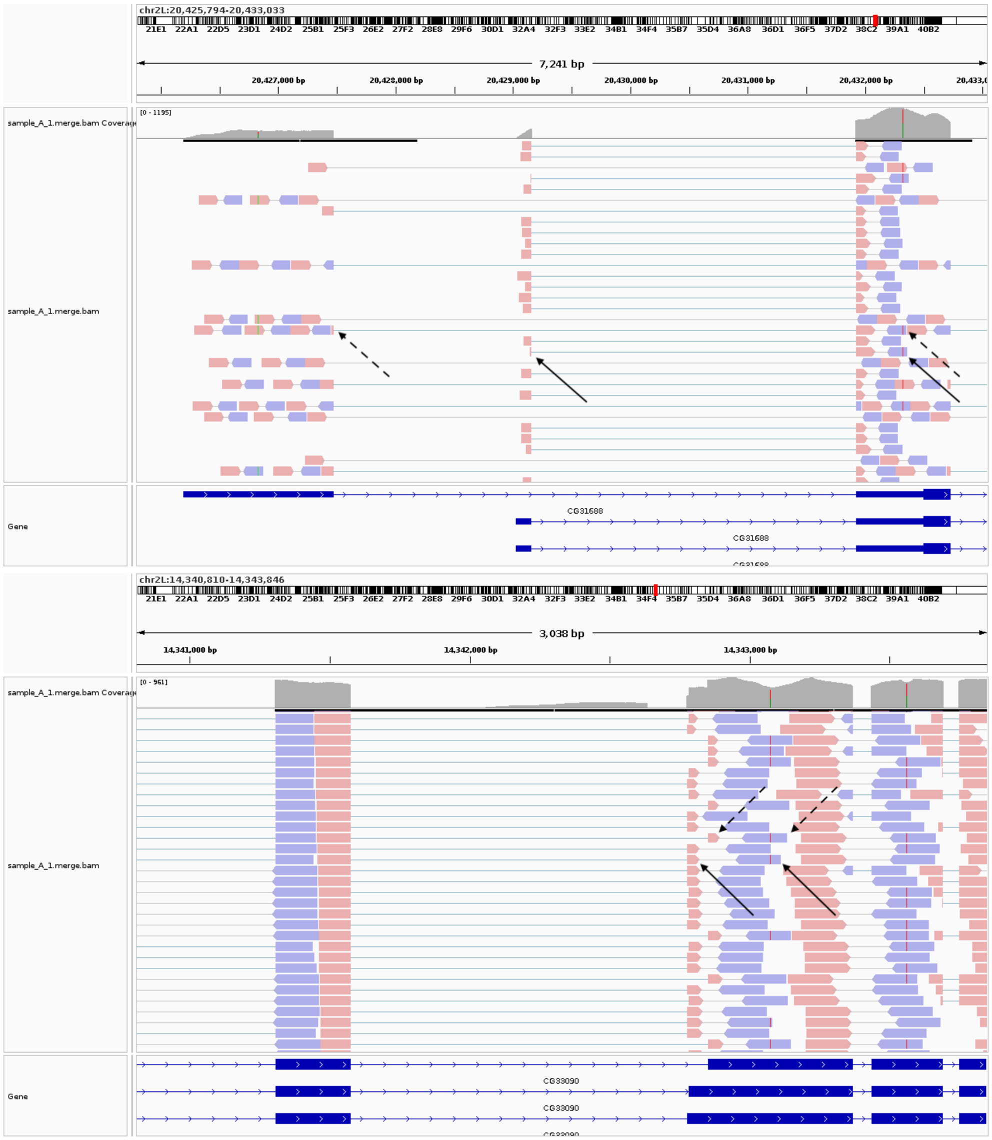

Figure S2: IGV visualization of HISAT2 aligned reads for the simulated *Drosophila melanogaster* dataset. The two depicted loci are *CG31688 / FBgn0263355* (top) and *CG33090 / FBgn0028916* (bottom). Paired-end reads such as those highlighted with dashed and solid arrows provide information to both isoform and allelic expression, used by *Salmon* to distribute isoform- and allelic-multi-mapping reads.

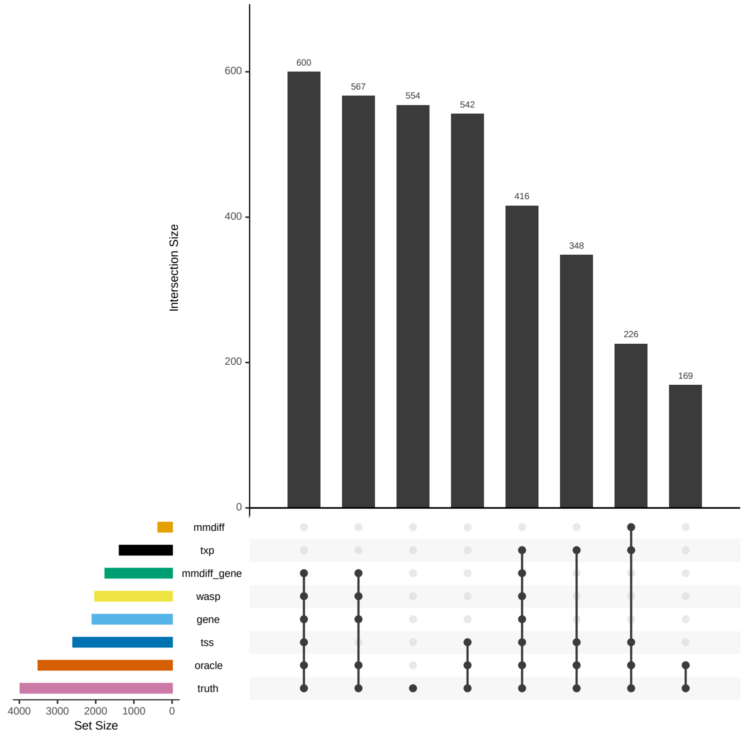

Figure S3: UpSet plot showing the overlap of simulation results at the transcript level comparing *SEESAW* at various levels of aggregation to *mmdiff* and *WASP*. The overlap is shown of different methods' positive sets as well as the true AI status of each transcript.

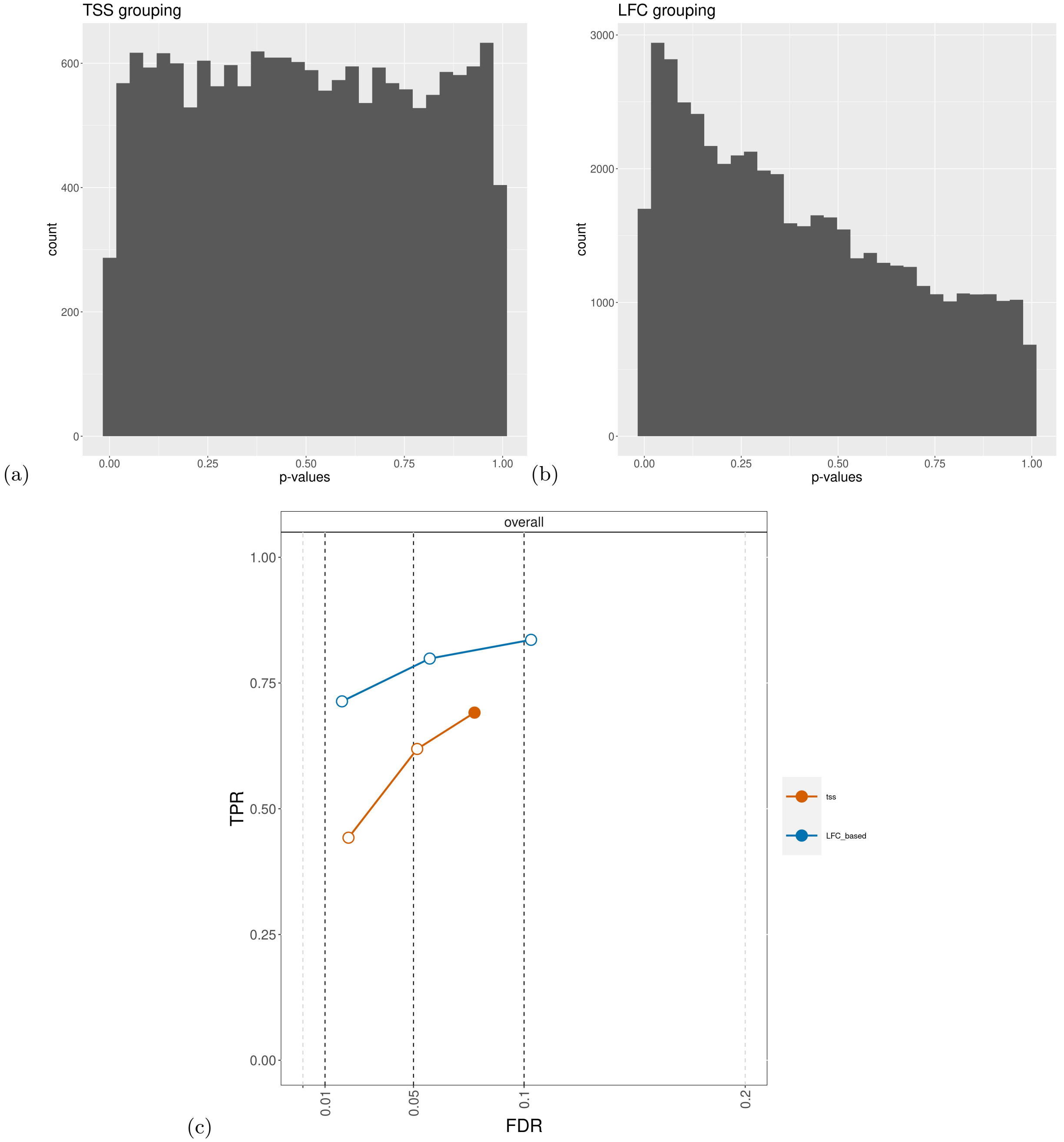

Figure S4: Simulation results comparing *SEESAW* with TSS grouping and log fold change (LFC) grouping. A) Distribution of p-values for transcripts under the null hypothesis of no allelic difference with TSS grouping. p-values were uniformly distributed as expected under the null. B) Distribution of p-values for transcripts under the null hypothesis with LFC grouping. p-values had a clear trend sloping up toward 0, and did not follow a uniform distribution. C) Overall sensitivity over false discovery rate for TSS aggregation (red) and LFC-based aggregation (blue). While LFC-based aggregation at 10% did not begin to show loss of FDR control, it could be expected for increasing sample size.

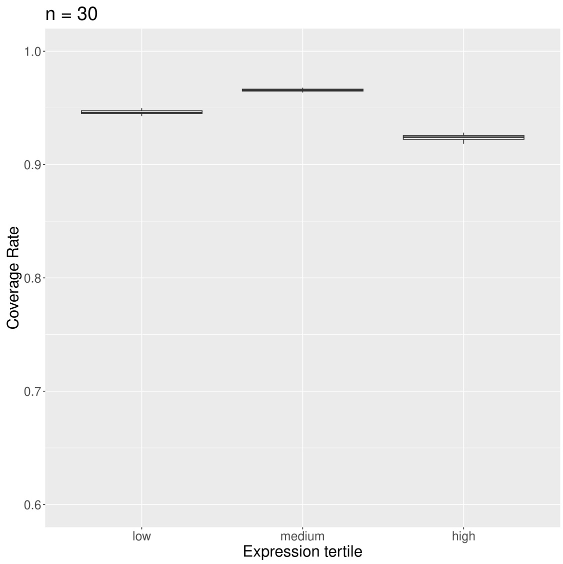

(a)

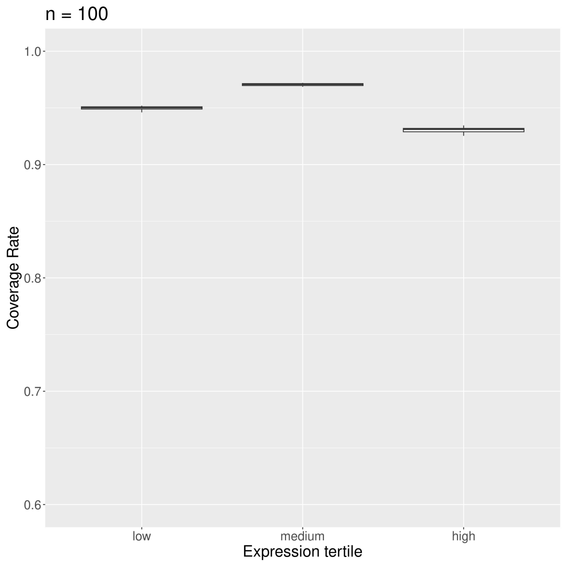

(b)

Figure S5: Bootstrap interval coverage rate across expression bins for A) $n = 30$ inferential replicates and B) $n = 100$ inferential replicates.

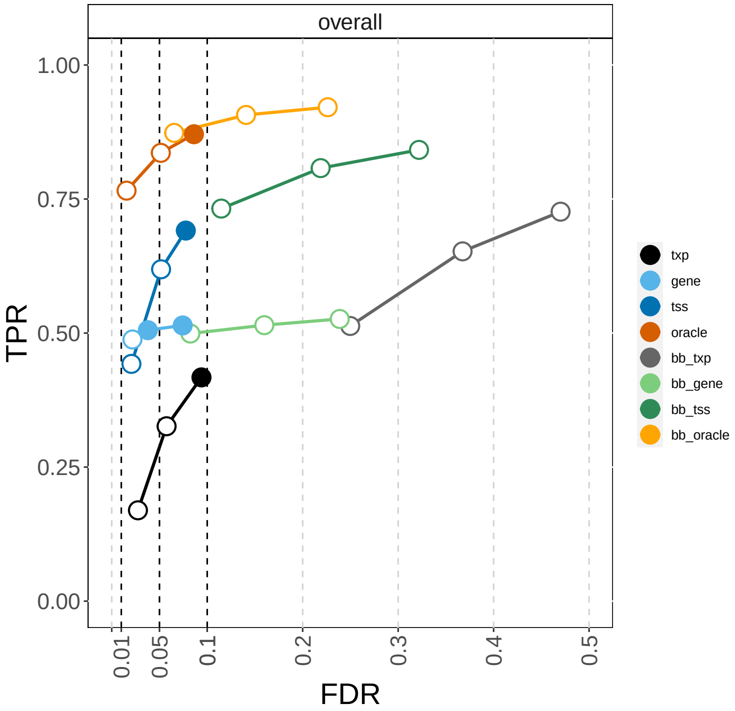

Figure S6: Simulation results comparing *SEESAW* at various levels of aggregation to a beta-binomial regression applied directly to estimated counts from *Salmon* at the transcript, TSS, gene, and oracle level. The beta-binomial regression does not make use of the inferential uncertainty.

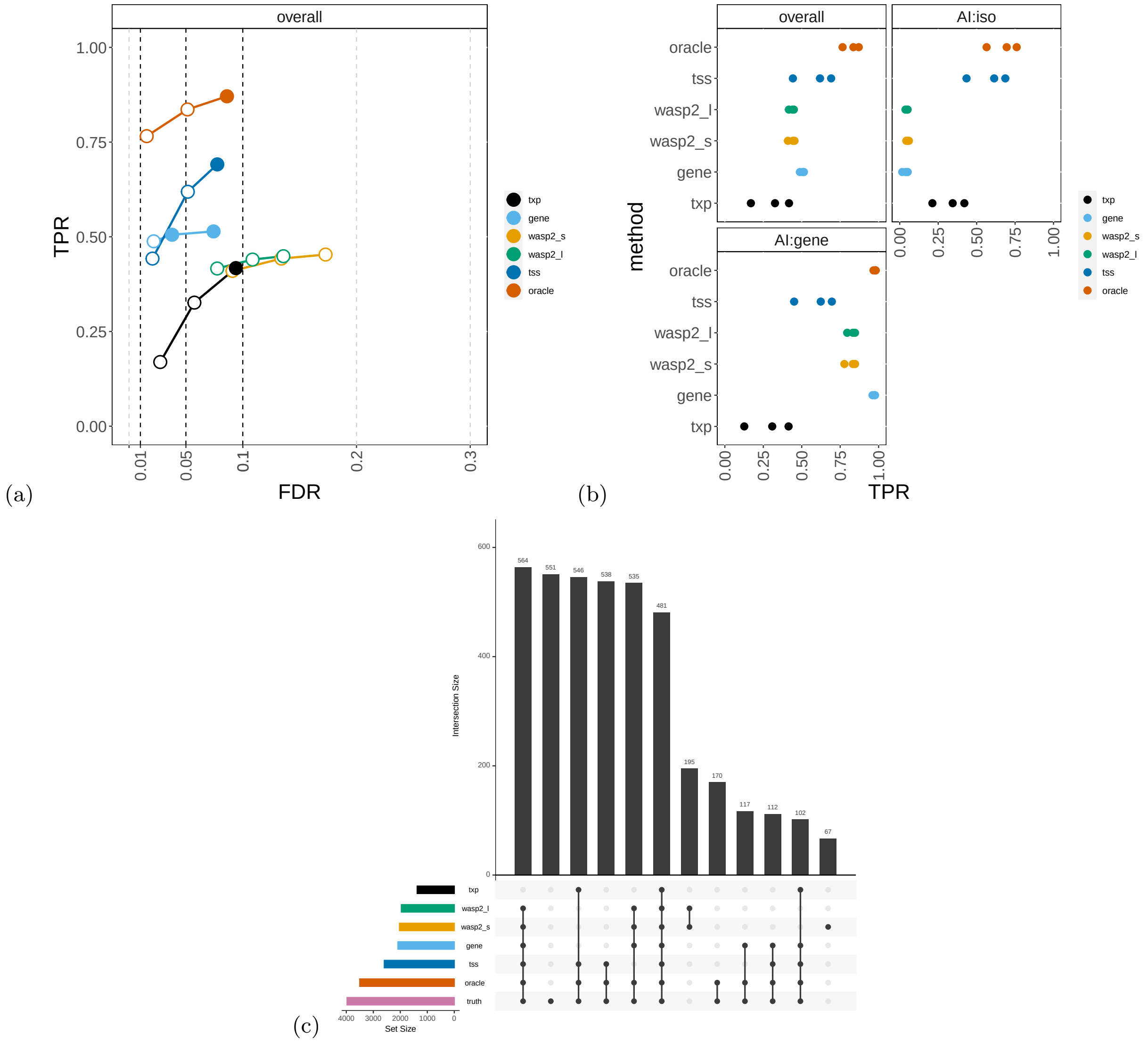

Figure S7: Simulation results at the transcript level comparing *SEESAW* at various levels of aggregation to *WASP2*. Both "single" (s) and "linear" (l) models in *WASP2* were assessed. A) Overall sensitivity over false discovery rate. B) Sensitivity overall and stratified by type of simulated AI. C) UpSet plot of the overlap of transcripts called by the methods and true AI transcripts.

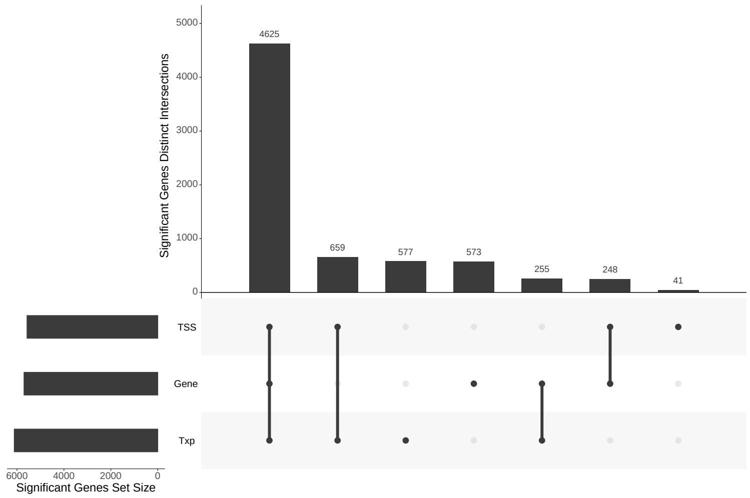

Figure S8: For the mouse osteoblast experiment, UpSet plot showing the overlap of significant genes, testing for global AI at various levels of aggregation using *SEESAW*.

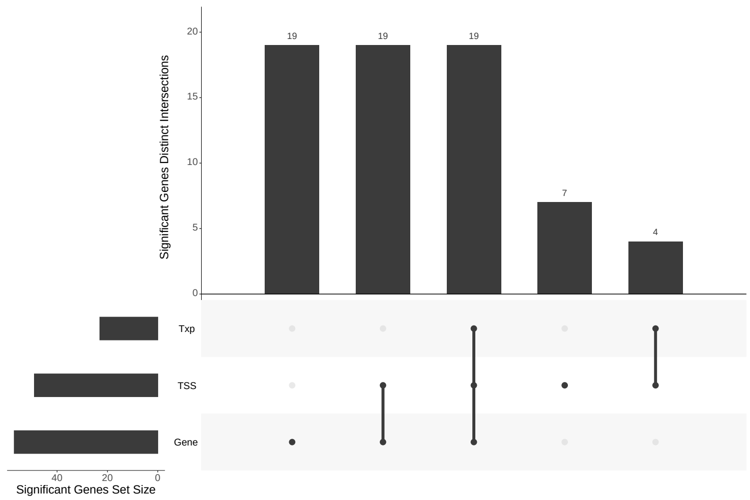

Figure S9: For the mouse osteoblast experiment, UpSet plot showing the overlap of significant genes, testing for dynamic AI at various levels of aggregation using *SEESAW*.

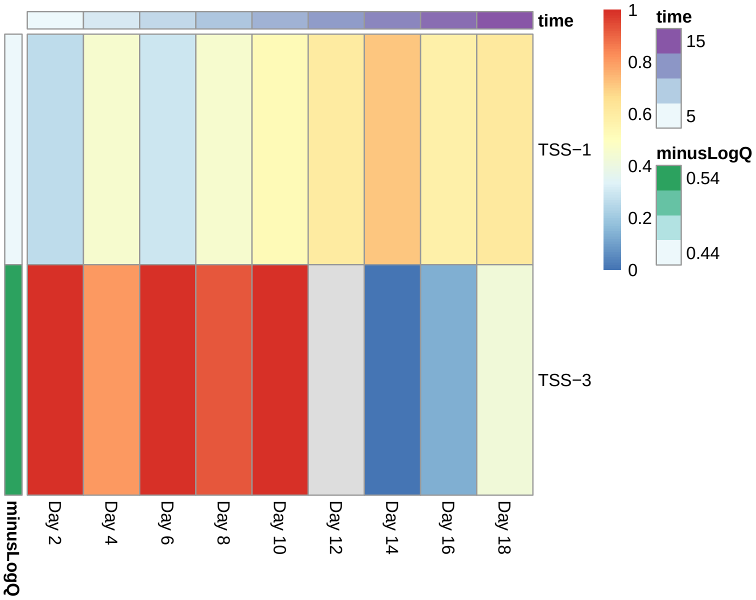

Figure S10: Allelic heatmap for two TSS groups of *Rasl11b*. minusLogQ denotes the $-\log_{10}(q\text{-value})$ for dynamic AI testing for each TSS group. Color indicates the fraction of total expression from the CAST/EiJ allele.

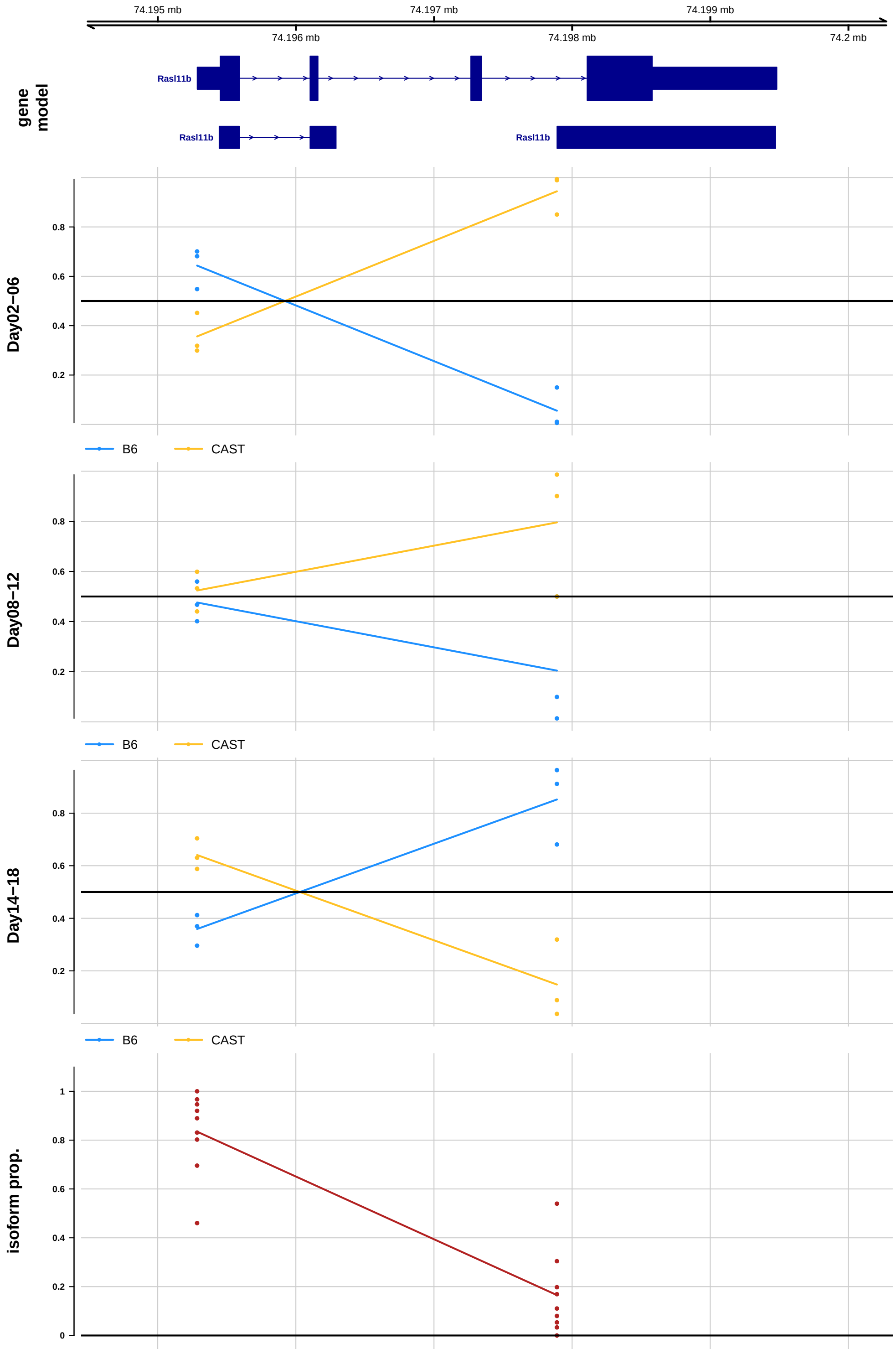

Figure S11: Gene model plot for *Rasl11b* at TSS-group level. Alelic ratios are shown over three grouped time points: day 2-6 (top row), day 8-12 (middle row) and day 14-18 (bottom row). Isoform proportions from all days shown in the fourth row.

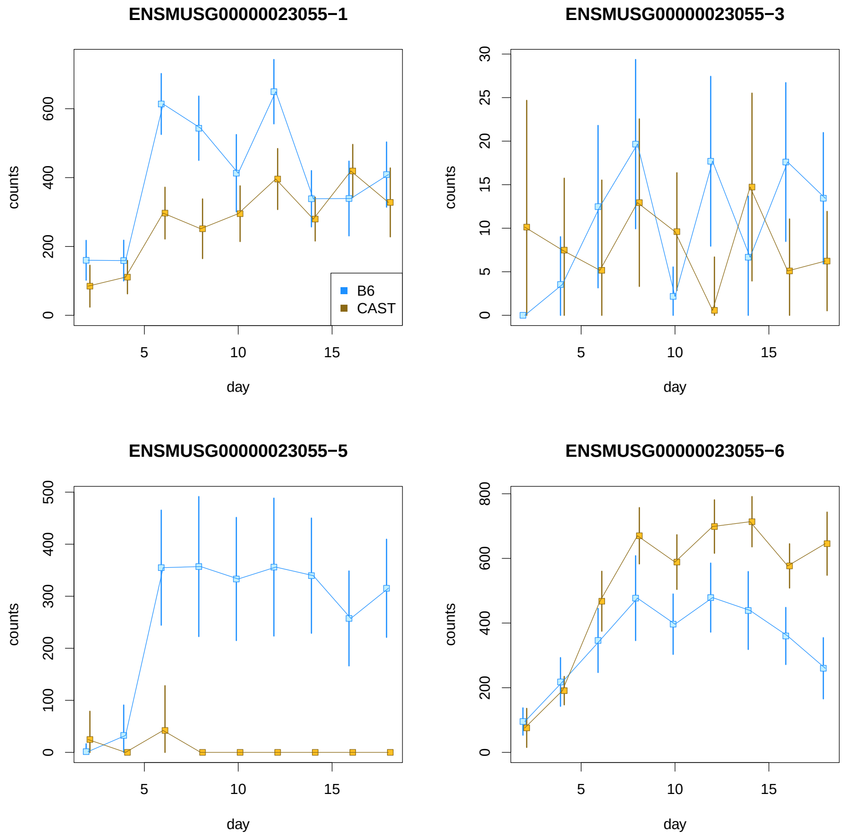

Figure S12: Estimated allelic counts over time for TSS groups of *Calcoco1*. Shown are four TSS groups of which "5" and "6" were significant for dynamic AI (FDR $< 5\%$). Estimation uncertainty shown with error bars (95% intervals based on bootstrap variance).

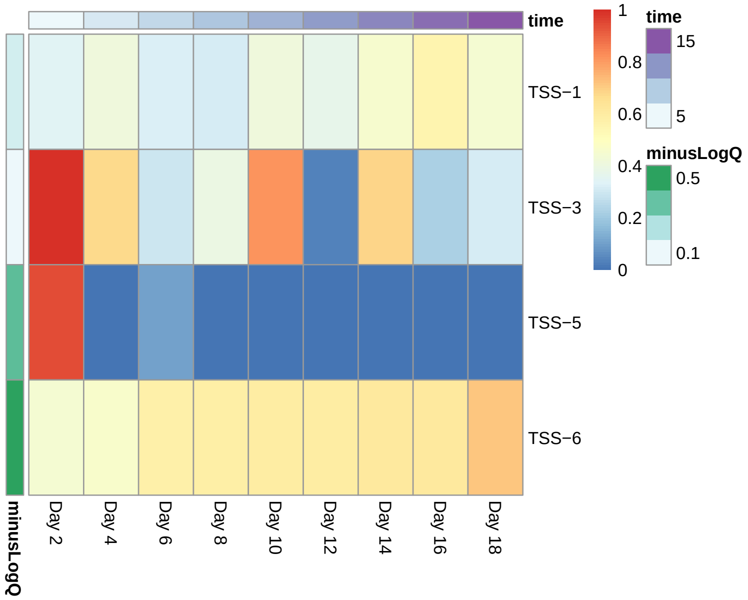

Figure S13: Allelic heatmap for four TSS groups of *Calcoco1*. minusLogQ denotes the $-\log_{10}(q\text{-value})$ for dynamic AI testing for each TSS group. Color indicates the fraction of total expression from the CAST/EiJ allele.

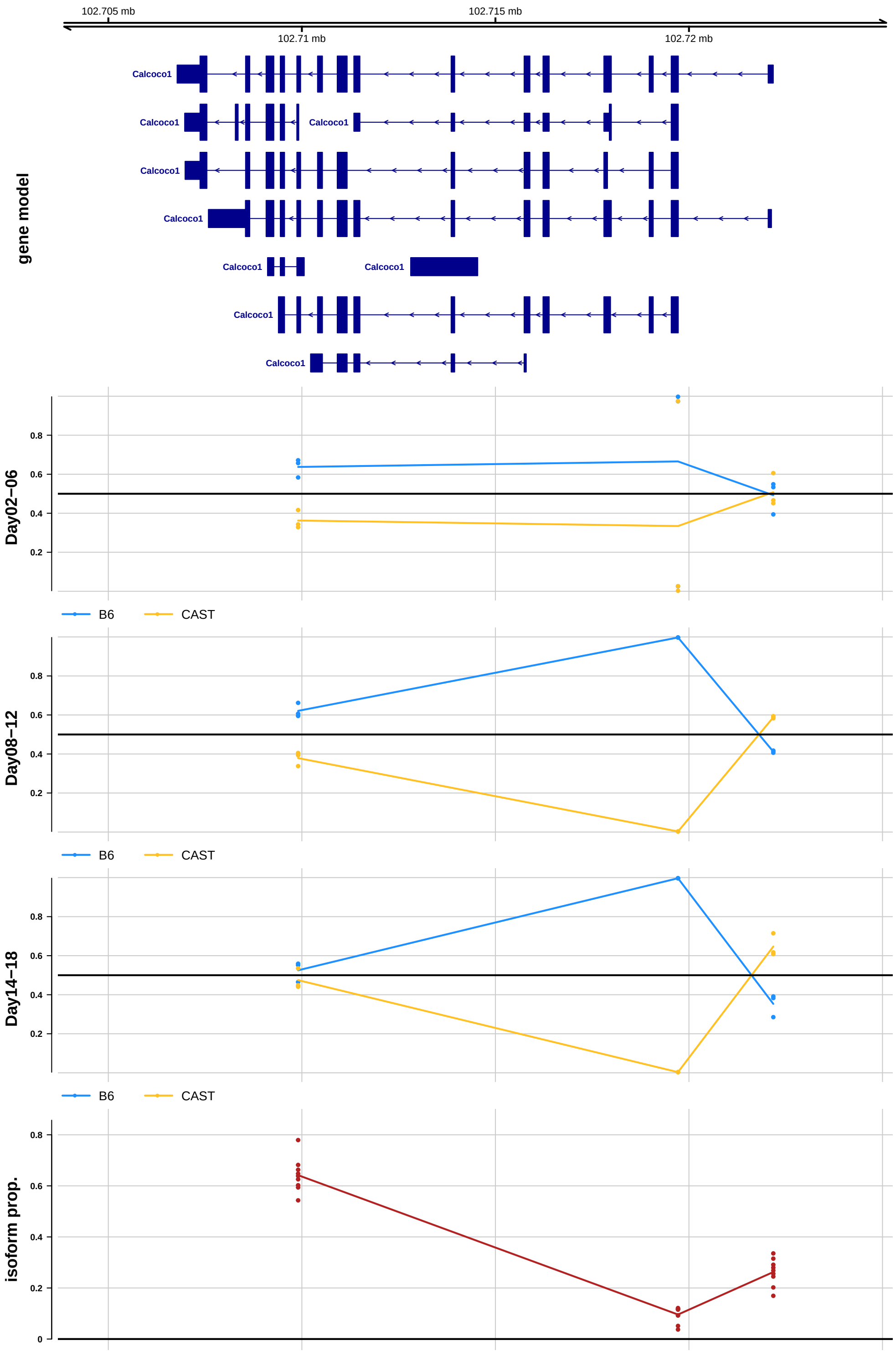

Figure S14: Gene model plot for *Calcoco1* at TSS-group level. Allelic ratios are shown over three grouped time points: day 2-6 (top row), day 8-12 (middle row) and day 14-18 (bottom row). Isoform proportions from all days shown in the fourth row. From left to right, the TSS groups are “1”, “5”, and “6”. TSS group “3” was filtered in this plot due to too low counts and isoform proportion.

	transcript			TSS			Gene		
	low	medium	high	low	medium	high	low	medium	high
infRep #10	.9040 (.0018)	.8953 (.0029)	.8295 (.0028)	.9333 (.0020)	.9448 (.0014)	.8988 (.0026)	.9466 (.0024)	.9673 (.0012)	.9158 (.0034)
infRep #20	.9171 (.0013)	.9163 (.0027)	.8515 (.0038)	.9434 (.0020)	.9611 (.0021)	.9185 (.0029)	.9550 (.0023)	.9774 (.0012)	.9300 (.0035)
infRep #30	.9209 (.0012)	.9226 (.0018)	.8578 (.0030)	.9462 (.0020)	.9656 (.0013)	.9241 (.0030)	.9568 (.0026)	.9801 (.0014)	.9338 (.0032)
infRep #50	.9243 (.0011)	.9270 (.0022)	.8623 (.0034)	.9485 (.0019)	.9683 (.0017)	.9279 (.0026)	.9585 (.0024)	.9816 (.0016)	.9367 (.0030)
infRep #100	.9261 (.0013)	.9300 (.0022)	.8660 (.0034)	.9498 (.0019)	.9706 (.0011)	.9307 (.0027)	.9594 (.0023)	.9826 (.0017)	.9384 (.0030)

Table S1: Assessment of bootstrap interval coverage. Mean (and standard deviation) of sample-wise coverage rate for each aggregation-level and number of inferential replicates. Number of "infRep" represents the number of inferential replicates (bootstraps) used to calculate the 95% bootstrap interval for the estimated count. Coverage is equal to 1 for a feature (transcript/TSS group/gene) and a sample if the true, simulated count falls within a 95% bootstrap interval using the bootstrap counts for that sample, and 0 otherwise. Features (transcript/TSS/gene) are divided into three categories by tertile of true counts.

			Gene		TSS	
	TSS group	Gene symbol	log2FC	*q*-value	log2FC	*q*-value
1	ENSMUSG00000000753-5	Serpinf1	-0.4972	0.0000	1.4195	0.0167
2	ENSMUSG00000001173-4	Ocrl	2.5080	0.0000	-0.7054	0.0015
3	ENSMUSG00000001175-2	Calm1	-0.2359	0.0000	2.3637	0.0327
4	ENSMUSG00000001794-6	Capns1	0.3873	0.0000	-0.1838	0.0362
5	ENSMUSG00000003033-4	Ap1m1	1.1178	0.0000	-1.2181	0.0411
6	ENSMUSG00000003131-2	Pafah1b2	-0.1783	0.0000	1.4019	0.0379
7	ENSMUSG00000003161-3	Sri	0.4347	0.0000	-0.2140	0.0295
8	ENSMUSG00000004415-5	Col26a1	0.4712	0.0079	-0.7012	0.0379
9	ENSMUSG00000005161-6	Prdx2	-0.7933	0.0000	2.6237	0.0006
10	ENSMUSG00000005846-2	Rsl1d1	0.2641	0.0020	-3.8551	0.0027
11	ENSMUSG00000006586-4	Runx1t1	-0.3607	0.0218	0.8016	0.0144
12	ENSMUSG00000006673-3	Qrich1	-0.5448	0.0000	0.4749	0.0362
13	ENSMUSG00000008348-2	Ubc	1.5110	0.0000	-0.4380	0.0073
14	ENSMUSG00000008682-2	Rpl10	-1.0812	0.0006	2.1585	0.0172
15	ENSMUSG00000017493-3	Igfbp4	1.6418	0.0000	-0.8379	0.0331
16	ENSMUSG00000017493-4	Igfbp4	1.6418	0.0000	-6.6867	0.0030
17	ENSMUSG00000017615-1	Tnfaip1	0.1248	0.0457	-7.4663	0.0000
18	ENSMUSG00000017778-1	Cox7c	0.2850	0.0000	-0.9979	0.0210
19	ENSMUSG00000018593-5	Sparc	-0.1406	0.0000	1.7404	0.0000
20	ENSMUSG00000019810-4	Fuca2	1.8390	0.0000	-5.9148	0.0000
21	ENSMUSG00000020027-1	Socs2	0.3111	0.0194	-1.9710	0.0000
22	ENSMUSG00000020053-3	Igf1	0.1153	0.0109	-2.1336	0.0137
23	ENSMUSG00000020658-5	Efr3b	-1.1261	0.0014	1.5989	0.0008
24	ENSMUSG00000020923-6	Ubtf	-0.5387	0.0012	3.6245	0.0373
25	ENSMUSG00000020935-3	Dcakd	0.5468	0.0000	-1.5434	0.0476
26	ENSMUSG00000021036-2	Sptlc2	0.2787	0.0003	-1.5458	0.0411
27	ENSMUSG00000021076-6	Actr10	0.9179	0.0000	-2.4966	0.0004
28	ENSMUSG00000021477-1	Ctsl	0.2398	0.0160	-6.4631	0.0004
29	ENSMUSG00000021484-1	Lman2	-1.0394	0.0000	1.4157	0.0214
30	ENSMUSG00000021670-5	Hmgcr	0.2182	0.0084	-1.2604	0.0048
31	ENSMUSG00000021728-3	Emb	-0.4907	0.0000	3.7344	0.0000
32	ENSMUSG00000021846-6	Peli2	-0.7951	0.0005	1.8248	0.0028
33	ENSMUSG00000021870-7	Slmap	-0.4729	0.0000	3.4371	0.0203
34	ENSMUSG00000022146-2	Osmr	0.3266	0.0000	-0.6113	0.0116
35	ENSMUSG00000022206-1	Npr3	0.6544	0.0000	-1.0981	0.0157
36	ENSMUSG00000022680-7	Pdxdc1	-0.7381	0.0000	0.9941	0.0000
37	ENSMUSG00000022969-1	Il10rb	1.1609	0.0000	-4.1329	0.0012
38	ENSMUSG00000022969-3	Il10rb	1.1609	0.0000	-1.3578	0.0290
39	ENSMUSG00000023055-6	Calcoco1	-0.3303	0.0237	0.4867	0.0254
40	ENSMUSG00000024066-4	Xdh	-0.6766	0.0032	3.3216	0.0020
41	ENSMUSG00000024085-3	Man2a1	0.3737	0.0000	-2.3129	0.0342
42	ENSMUSG00000024535-2	Snx24	0.4824	0.0008	-1.4142	0.0435
43	ENSMUSG00000024576-4	Csnk1a1	0.5243	0.0000	-0.5855	0.0203
44	ENSMUSG00000025133-5	Ints4	-0.4504	0.0000	1.3647	0.0135
45	ENSMUSG00000025393-2	Atp5b	-0.1393	0.0003	1.3180	0.0000
46	ENSMUSG00000025451-3	Paip1	0.5420	0.0010	-2.5358	0.0148
47	ENSMUSG00000025933-2	Tmem14a	0.5184	0.0354	-2.5237	0.0012
48	ENSMUSG00000026185-1	Igfbp5	0.3399	0.0148	-5.5603	0.0091
49	ENSMUSG00000026193-2	Fn1	0.7881	0.0000	-3.6801	0.0163
50	ENSMUSG00000026193-5	Fn1	0.7881	0.0000	-0.6366	0.0206

51	ENSMUSG00000026193-6	Fn1	0.7881	0.0000	-0.9125	0.0015
52	ENSMUSG00000026335-3	Pam	-0.1421	0.0304	1.5230	0.0073
53	ENSMUSG00000026335-9	Pam	-0.1421	0.0304	0.5550	0.0016
54	ENSMUSG00000026399-2	Cd55	1.7062	0.0000	-2.2590	0.0246
55	ENSMUSG00000026728-2	Vim	-0.2921	0.0000	2.2703	0.0238
56	ENSMUSG00000027201-1	Myef2	0.4134	0.0015	-1.7408	0.0406
57	ENSMUSG00000027272-5	Ubr1	0.4043	0.0015	-1.2831	0.0144
58	ENSMUSG00000027523-4	Gnas	0.6816	0.0008	-2.7420	0.0011
59	ENSMUSG00000027663-2	Zmat3	0.5589	0.0000	-5.9277	0.0088
60	ENSMUSG00000027750-2	Postn	-0.2160	0.0000	2.9264	0.0000
61	ENSMUSG00000027750-4	Postn	-0.2160	0.0000	0.7632	0.0122
62	ENSMUSG00000027797-8	Dclk1	0.3300	0.0005	-0.9146	0.0030
63	ENSMUSG00000027852-3	Nras	0.3541	0.0000	-1.1067	0.0430
64	ENSMUSG00000028114-3	Mettl14	-0.4781	0.0026	1.7074	0.0430
65	ENSMUSG00000028234-3	Rps20	0.1662	0.0011	-0.1665	0.0004
66	ENSMUSG00000028552-2	Eps15	0.3109	0.0000	-3.3418	0.0148
67	ENSMUSG00000028936-5	Rpl22	-0.2391	0.0006	1.0503	0.0457
68	ENSMUSG00000029061-3	Mmp23	0.1041	0.0421	-2.3856	0.0000
69	ENSMUSG00000029068-2	Ccnl2	0.3023	0.0334	-1.0018	0.0135
70	ENSMUSG00000029098-4	Acox3	0.4384	0.0140	-1.2028	0.0140
71	ENSMUSG00000029098-5	Acox3	0.4384	0.0140	-1.6052	0.0395
72	ENSMUSG00000029131-5	Dnajb6	-2.1779	0.0000	1.7201	0.0210
73	ENSMUSG00000029190-4	D5Ertd579e	0.3623	0.0017	-1.2681	0.0267
74	ENSMUSG00000029213-2	Commd8	-0.3497	0.0025	1.3324	0.0319
75	ENSMUSG00000029657-5	Hsph1	-0.2035	0.0185	0.7964	0.0006
76	ENSMUSG00000029802-1	Abcg2	-0.5457	0.0030	0.8937	0.0034
77	ENSMUSG00000029815-2	Malsu1	-1.5641	0.0000	2.7576	0.0036
78	ENSMUSG00000029994-3	Anxa4	0.2721	0.0003	-2.5104	0.0000
79	ENSMUSG00000030243-3	Recql	-0.9194	0.0011	1.3294	0.0097
80	ENSMUSG00000030417-1	Pdcd5	0.5639	0.0010	-1.1299	0.0494
81	ENSMUSG00000030532-3	Hddc3	1.3584	0.0000	-2.9718	0.0027
82	ENSMUSG00000030770-1	Parva	0.2879	0.0000	-5.5121	0.0015
83	ENSMUSG00000030770-4	Parva	0.2879	0.0000	-0.5458	0.0196
84	ENSMUSG00000030881-1	Arfip2	-0.5348	0.0039	2.5773	0.0390
85	ENSMUSG00000031422-1	Morf4l2	-8.4574	0.0000	1.3739	0.0137
86	ENSMUSG00000031600-4	Vps37a	0.3284	0.0011	-0.6672	0.0314
87	ENSMUSG00000031813-3	Mvb12a	0.5846	0.0003	-1.6786	0.0034
88	ENSMUSG00000031902-4	Nfatc3	-5.7483	0.0000	2.8369	0.0000
89	ENSMUSG00000033685-3	Ucp2	0.4107	0.0035	-1.5758	0.0304
90	ENSMUSG00000034342-7	Cbl	0.4647	0.0000	-3.4540	0.0196
91	ENSMUSG00000035171-5	1110059E24Rik	0.5027	0.0000	-1.8531	0.0116
92	ENSMUSG00000035621-5	Midn	-0.4541	0.0006	2.3960	0.0088
93	ENSMUSG00000036391-2	Sec24a	-0.1624	0.0308	2.0602	0.0417
94	ENSMUSG00000036775-2	Decr2	-1.7122	0.0000	2.1186	0.0000
95	ENSMUSG00000037805-3	Rpl10a	1.3370	0.0000	-1.9229	0.0012
96	ENSMUSG00000037822-4	Smim14	0.1496	0.0183	-0.8618	0.0000
97	ENSMUSG00000038072-1	Galnt11	0.3297	0.0079	-1.2292	0.0000
98	ENSMUSG00000038776-1	Ephx1	0.6239	0.0000	-3.6039	0.0058
99	ENSMUSG00000039234-6	Sec24d	0.1521	0.0040	-3.7032	0.0006
100	ENSMUSG00000040118-3	Cacna2d1	0.3744	0.0026	-1.2351	0.0304

101	ENSMUSG00000040118-4	Cacna2d1	0.3744	0.0026	-3.1706	0.0000
102	ENSMUSG00000040296-2	Ddx58	-0.3435	0.0000	1.4863	0.0004
103	ENSMUSG00000040667-8	Nup88	-0.1958	0.0325	1.1224	0.0347
104	ENSMUSG00000040760-2	Appl1	0.2833	0.0109	-1.0734	0.0012
105	ENSMUSG00000041272-4	Tox	0.8936	0.0000	-2.9804	0.0100
106	ENSMUSG00000041623-2	D11Wsu47e	0.4674	0.0218	-1.4240	0.0206
107	ENSMUSG00000041638-2	Gcn1	0.1861	0.0446	-2.3530	0.0011
108	ENSMUSG00000041702-1	Btbd7	-0.3193	0.0000	0.9751	0.0043
109	ENSMUSG00000041926-7	Rnpep	-0.7554	0.0010	1.4793	0.0036
110	ENSMUSG00000042426-2	Dhx29	-0.5292	0.0000	2.3293	0.0000
111	ENSMUSG00000042472-7	Zfp410	-0.4347	0.0463	0.9386	0.0357
112	ENSMUSG00000042712-1	Tceal9	-0.6166	0.0358	2.5494	0.0000
113	ENSMUSG00000043091-2	Tuba1c	1.0560	0.0000	-2.0684	0.0242
114	ENSMUSG00000045867-2	Cradd	-0.9538	0.0044	2.3222	0.0091
115	ENSMUSG00000046463-3		0.8324	0.0000	-1.5492	0.0441
116	ENSMUSG00000047909-6	Ankrd16	0.3240	0.0262	-1.2854	0.0275
117	ENSMUSG00000049470-6	Aff4	0.1891	0.0011	-4.0426	0.0018
118	ENSMUSG00000049470-7	Aff4	0.1891	0.0011	-1.8730	0.0342
119	ENSMUSG00000049775-1	Tmsb4x	-2.1537	0.0013	1.9639	0.0020
120	ENSMUSG00000051007-1	Gatd1	0.4867	0.0027	-1.3180	0.0390
121	ENSMUSG00000052146-4	Rps10	0.1214	0.0215	-0.1761	0.0148
122	ENSMUSG00000052353-2	Cemip	-0.4288	0.0000	0.4442	0.0030
123	ENSMUSG00000052406-4	Rexo4	-1.0186	0.0003	1.6406	0.0050
124	ENSMUSG00000052920-5	Prkg1	-0.5598	0.0000	3.4466	0.0012
125	ENSMUSG00000053453-4	Thoc7	0.3225	0.0027	-1.7004	0.0050
126	ENSMUSG00000054452-6	Tle5	0.3549	0.0000	-2.8575	0.0230
127	ENSMUSG00000055301-3	Adh7	1.4580	0.0000	-1.9470	0.0263
128	ENSMUSG00000056167-3	Cnot10	-0.2406	0.0262	1.7613	0.0106
129	ENSMUSG00000056536-3	Pign	0.4257	0.0008	-1.6103	0.0024
130	ENSMUSG00000060227-2	Golm2	-0.3406	0.0030	0.7822	0.0018
131	ENSMUSG00000060510-1	Zfp266	0.2050	0.0019	-1.3106	0.0337
132	ENSMUSG00000061477-1	Rps7	-0.6803	0.0000	2.2734	0.0088
133	ENSMUSG00000062604-1	Srpk2	0.2478	0.0344	-1.3658	0.0012
134	ENSMUSG00000062604-4	Srpk2	0.2478	0.0344	-0.7159	0.0481
135	ENSMUSG00000063087-2	Gm10125	-1.3520	0.0029	1.4983	0.0091
136	ENSMUSG00000063524-6	Eno1	-0.6924	0.0000	5.4608	0.0011
137	ENSMUSG00000066415-1	Msl2	0.6177	0.0015	-1.8527	0.0122
138	ENSMUSG00000068566-2	Myadm	0.1248	0.0099	-0.5123	0.0004
139	ENSMUSG00000068823-5	Csde1	-0.2529	0.0204	3.8378	0.0045
140	ENSMUSG00000070469-2	Adamtsl3	-0.3452	0.0201	2.9881	0.0177
141	ENSMUSG00000070942-3	Il1rl2	-1.3181	0.0000	2.5847	0.0014
142	ENSMUSG00000073530-5	Pappa2	-0.2977	0.0023	0.7394	0.0238
143	ENSMUSG00000074466-3	Gm15417	1.0034	0.0115	-1.0393	0.0367
144	ENSMUSG00000092341-2	Malat1	-0.5146	0.0000	3.4349	0.0226
145	ENSMUSG00000097073-2	9430037G07Rik	-1.1082	0.0198	1.4376	0.0435

Table S2: Genes that exhibited global AI with discordant direction of AI at gene and TSS-group level. Each row represents a TSS group that was found significant for the global AI test (FDR $< 5\%$) and which had discordant sign compared to the gene-level AI (also FDR $< 5\%$).
