# Supplementary material for: SEESAW: detecting isoform-level allelic imbalance accounting for inferential uncertainty
Source: Genome Biol. 2023 Jul 12;24:165. doi: 10.1186/s13059-023-03003-x (PMC10337143; doi:10.1186/s13059-023-03003-x)
Supplement: Supplementary file 2 — Additional file 2. Review history. [file 13059_2023_3003_MOESM2_ESM.docx]

**1^st^ round**

**Reviewer 1**

The authors proposed a suite of methods, SEESAW, to quantify the AI at the allele, isoform, and gene level, and also to perform statistical test for global and dynamic AI. The pipeline provided by the authors can be a very useful toolset for researchers who are interested in doing comprehensive RNAseq-driven quantitative studies, particularly, the allelic gene expression. Overall the statistical methods are reasonable, and the manuscript is well written. I have several major questions/concerns about this manuscript as follows:

1. Although the Methods section has provided a nice description of the approach, I still think a figure (or multiple) would help the audience better understand the approach, e.g., how to aggregate isoform expression (strict vs. fuzzy), and gene-level aggregation, etc.

2. The authors used bootstrap (~30) to capture the inferential uncertainty across genes. Can you please justify the variation generated by the bootstrap (and the number of bootstraps) is similar to the inferential uncertainty? Also please comment on whether your approach is robust to the number of bootstraps.

3. In real data analysis, it's unknown whether the AI of the isoforms is concordant or discordant within a gene. The power of detecting global AI at the gene level will be reduced for the discordant case. I'm wondering if it's possible to align the directions of the AI among the isoforms before aggregation at the gene level?

4. When testing the dynamic AI, for example, whether and how AI changes over time, the author proposed using the correlation coefficient (Spearman or Pearson), and using permutation to assess the significance of the correlation. I'm wondering if it's more flexible to put this in a regression framework? So that 2 or more covariates can be tested as well as the interaction? For example, the clinical condition and time, and whether the change in AI over time differs between conditions?

**Reviewer 2**

This manuscript addresses a critical yet challenging task: detecting allelic imbalance (AI). The authors presents SEESAW, a pipeline that is able to detect AIs at various levels (gene-level, isoform-level, and grouped TSS-level). SEESAW can also detect AIs that are consistent across all samples, differential AI across two groups of samples, and dynamic AI over a covariate (e.g., time). The major novelty of SEESAW lies in grouping transcripts with the same (or nearby) TSS together. With experimental studies the authors showed that such aggregation reduces inferential uncertainty and helps recover signal that would be lost if only testing at the isoform level. The resulting pipeline is available in fishpond R package. Overall the methods are interesting and the SEESAW adds a valuable tool for the community to study gene regulations with RNA-seq data.

My concerns are:

1, SEESAW significantly outperforms other methods on detecting discordant AIs but on concordant AIs the improvement seems marginal. As the experiments are done with simulations, it is not clear what is the biological significance of detecting discordant AIs. The authors should address this by referring to the literature, and/or apply SEESAW to detect/analyze discordant AIs on biological data.

2, SEESAW uses Salmon to perform allele-specific, isoform-level quantification. The problem seems very challenging as most reads don't overlap with variants. It would be helpful to illustrate the accuracy of quantification and how it affects the downstream differential analysis (by Swish).

3, In Figure 2 it was not clear what discordant/concordant AIs, oracles, etc, mean. They were explained later in the Methods but should be also mentioned in the figure caption or in the main text to ease reading. Similar situation for Figure 3: how the "isoform proportion" was calculated? What B6, CAST, and "counts" mean in (B) and (C)? These unexplained terms interrupt the reading flow and could be confusing for readers.

**Authors’ response**

Reviewer #1: The authors proposed a suite of methods, SEESAW, to quantify the AI at the allele, isoform, and gene level, and also to perform statistical test for global and dynamic AI. The pipeline provided by the authors can be a very useful toolset for researchers who are interested in doing comprehensive RNAseq-driven quantitative studies, particularly, the allelic gene expression. Overall the statistical methods are reasonable, and the manuscript is well written. I have several major questions/concerns about this manuscript as follows:

1. Although the Methods section has provided a nice description of the approach, I still think a figure (or multiple) would help the audience better understand the approach, e.g., how to aggregate isoform expression (strict vs. fuzzy), and gene-level aggregation, etc.

We have added a new Figure 1 that gives a more clear overview of the approach of aggregation followed by testing, and have moved the diagram of the SEESAW pipeline to Supp Figure 1.

We have further clarified the sentence introducing "fuzzy" aggregation on line 177:

"To facilitate data visualization, strengthen biological signal, and reduce inferential uncertainty, we grouped any transcripts with TSS within 50 bp of each other (referred to here as 'fuzzy TSS groups', to contrast with strict basepair-resolution TSS grouping)."

2. The authors used bootstrap (~30) to capture the inferential uncertainty across genes. Can you please justify the variation generated by the bootstrap (and the number of bootstraps) is similar to the inferential uncertainty? Also please comment on whether your approach is robust to the number of bootstraps.

We thank the reviewer for highlighting this important element of differential analysis when taking into account uncertainty of quantification. The uncertainty is measured per allele, per feature and per sample. The original bootstrap counts that are output by Salmon are provided on the transcript level, but our newly developed functionality, importAllelicCounts(), facilitates aggregating the bootstraps and the inferential uncertainty to higher levels (e.g. TSS- or gene-level aggregation) per sample.

We have performed a number of experiments that address the following questions:

● Is n=30 enough bootstraps to capture the inferential uncertainty?

● Do the bootstraps provide sufficient coverage of true count values in simulation?

● Does bootstrap interval coverage change across expression level or aggregation level?

We have included these new results in the Methods and Results sections, e.g., starting on line 148:

"As SEESAW makes use of Salmon for quantification, with inferential uncertainty measured via bootstraps, we evaluated the accuracy of uncertainty estimation using n = 10, 20, 30, 50 and 100 bootstraps, respectively to define 95% bootstrap intervals for the estimated counts…"

We find that n=30 bootstraps does provide sufficient inferential uncertainty for coverage, while not adding too much to compute or storage costs.

3. In real data analysis, it's unknown whether the AI of the isoforms is concordant or discordant within a gene. The power of detecting global AI at the gene level will be reduced for the discordant case. I'm wondering if it's possible to align the directions of the AI among the isoforms before aggregation at the gene level?

We agree with the reviewer that in advance we will not know the fraction of concordant or discordant AI in real datasets, and as we saw in the mouse osteoblast differentiation time course, the majority of genes may show concordant AI across isoforms. Additionally, it is true that the power may be reduced in the case that we do not decide a priori on the best grouping strategy. TSS-based aggregation proposed here was motivated by the fact that it is an independent feature from the read data, and that promoters may be subject to differential gene regulatory mechanisms (e.g. having variable promoter sequence and chromatin structure, or making contact with different enhancers).

It is conceptually attractive to perform data-driven aggregation, but doing so in a way that controls error is currently beyond the scope of this work. We have performed an additional experiment to demonstrate why we cannot currently recommend grouping based on the direction of the AI. In this experiment, we grouped the isoforms within a gene based on their sign of allelic log fold change (LFC) and then performed differential AI testing on the LFC-based groups. We showed that this approach lost error control as would be expected with the “double dipping” of data across samples. The p-values for the transcripts that were generated under the null hypothesis no longer followed a uniform distribution. We have included these results in the Methods and Results sections, e.g. starting on line 142:

“We demonstrated that error control was lost if we used allelic log fold change (LFC) in the aggregation step as described in Methods (Figure S7)...”

4. When testing the dynamic AI, for example, whether and how AI changes over time, the author proposed using the correlation coefficient (Spearman or Pearson), and using permutation to assess the significance of the correlation. I'm wondering if it's more flexible to put this in a regression framework? So that 2 or more covariates can be tested as well as the interaction? For example, the clinical condition and time, and whether the change in AI over time differs between conditions?

It is possible, as the reviewer points out, for the methods to handle generic designs outside of what is currently implemented within the swish() function: global AI, two-group differential AI, or dynamic AI along a continuous covariate.

To address this possibility and support further designs, we have added a new section to the allelic analysis vignette in the fishpond package, "More complex designs", where we describe how the Swish (and originally SAMseq) framework for nonparametric testing, can be extended.

We demonstrate using the example of two group differential correlation of AI with a continuous covariate, using a simple simulated dataset from the fishpond package. This is now described in the Discussion and Methods, e.g. from line 338:

"Additional tests can also be performed, following the Swish framework of averaging test statistics over bootstrap replicates and performing permutations to compute the q-values. An example of testing for differences in correlation of AI with a continuous covariate across two groups using a general regression framework is demonstrated in the software vignette for allelic analysis, under the heading, 'More complex designs'." A link to the new section of the allelic analysis vignette is here: https://mikelove.github.io/fishpond/articles/allelic.html#more-complex-designs

~~~

Reviewer #2: This manuscript addresses a critical yet challenging task: detecting allelic imbalance (AI). The authors present SEESAW, a pipeline that is able to detect AIs at various levels (gene-level, isoform-level, and grouped TSS-level). SEESAW can also detect AIs that are consistent across all samples, differential AI across two groups of samples, and dynamic AI over a covariate (e.g., time). The major novelty of SEESAW lies in grouping transcripts with the same (or nearby) TSS together. With experimental studies the authors showed that such aggregation reduces inferential uncertainty and helps recover signal that would be lost if only testing at the isoform level. The resulting pipeline is available in fishpond R package. Overall the methods are interesting and the SEESAW adds a valuable tool for the community to study gene regulations with RNA-seq data.

1, SEESAW significantly outperforms other methods on detecting discordant AIs but on concordant AIs the improvement seems marginal. As the experiments are done with simulations, it is not clear what is the biological significance of detecting discordant AIs. The authors should address this by referring to the literature, and/or apply SEESAW to detect/analyze discordant AIs on biological data.

In our original submission, we applied SEESAW to a novel dataset: an osteoblast time course from an F1 mouse cross, with nine time points between day 2 and day 18, finding 134 genes with discordant AI for the global AI test. After having reviewed the list for biological relevance to the osteoblast, we have included a new figure highlighting a gene showing discordant AI, Sparc, which is a key gene in bone development.

The Sparc example is included starting at line 195:

“Another gene of the 134 genes with discordant pattern was Sparc, the most highly expressed gene at the last time point in the osteoblast differentiation time course. Sparc is known to be critical for bone development….”

2, SEESAW uses Salmon to perform allele-specific, isoform-level quantification. The problem seems very challenging as most reads don't overlap with variants. It would be helpful to illustrate the accuracy of quantification and how it affects the downstream differential analysis (by Swish).

Indeed, only some fraction of the reads – varying across isoforms and genes – will overlap exonic variants and so provide information about allelic imbalance. This fraction could be low for transcripts that differ across alleles by only a single nucleotide, for example. As we use Salmon to perform quantification, the information from all types of reads (as diagrammed in a new Figure 1) is aggregated to inform the allelic estimates. By bootstrapping, it is revealed how much the estimated counts vary due to sampling of reads, where the variance depends on where the reads fall with respect to isoform- and allele-identifying transcript sequence.

Furthermore, if the bootstrap data is aggregated to higher levels of resolution, the information about the uncertainty is updated, with the general pattern being reduction in uncertainty at higher levels of resolution. Also in response to Reviewer 1, we have now included analysis showing that the uncertainty quantification by bootstrap is well calibrated (see response to Reviewer 1, comment #2 above).

Additionally, we have assessed how the use of estimated counts (with uncertainty) affects downstream analysis by Swish, by comparing the use of Swish to another typical AI testing method, beta-binomial GLM, which does not make use of the uncertainty of estimation of allele-specific, isoform-level counts. The approach not making use of inferential uncertainty (beta-binomial GLM) was not able to maintain control of FDR, across all of the different aggregation levels considered in the paper. In contrast, WASP uses only the observed counts (not estimated counts) of reads overlapping a particular SNP, after correcting for bias due to genomic multi-mapping, in its tests of allelic imbalance.

Our new analysis comparing SEESAW to the beta-binomial GLM applied to Salmon estimated counts is described in the Methods and Results, e.g. staring on line 159:

"We assessed if the use of inferential uncertainty by Swish resulted in better error control compared to AI analysis with a beta-binomial generalized linear model…"

We therefore recommend running Swish downstream of allelic quantification with Salmon as it protects against potential loss of FDR control when inferential uncertainty is high, as seen previously in differential transcript expression (DTE) analyses (Zhu 2019).

3, In Figure 2 it was not clear what discordant/concordant AIs, oracles, etc, mean. They were explained later in the Methods but should be also mentioned in the figure caption or in the main text to ease reading. Similar situation for Figure 3: how the "isoform proportion" was calculated?

What B6, CAST, and "counts" mean in (B) and (C)? These unexplained terms interrupt the reading flow and could be confusing for readers.

We thank the reviewer for highlighting that these terms, when introduced without context may hinder the readability of the manuscript. We have further explained these terms as they occur in the main text and captions.

As a summary we provide the table below that explains these terms:

| Fig.2 | Discordant AI | "Discordant AI refers to the  case where isoforms within a  gene have opposite  directions of AI, while  concordant AI refers to the  case where isoforms within a  gene have the same direction  of AI." |
| --- | --- | --- |
|  | Concordant AI |  |
|  | txp/tss/gene | "SEESAW was applied at  different levels of resolution  including transcript (txp),  aggregated-to-TSS (TSS),  aggregated-to-gene (gene)..." |
|  | oracle | "...where oracle involved  aggregating transcripts by the  true AI signal direction,  known only in simulation" |
| Fig. 3 | B6 | C57BL/6J |
|  | CAST | CAST/EiJ |
|  | Isoform proportion | Isoform proportion per TSS  group was calculated by  summing the estimated TPM  (transcript per million) of the  isoforms in the group and  dividing by the gene-level  TPM. |
|  | Allelic proportion | Allelic proportion was  calculated by dividing  estimated allelic counts for  each strain by the total  counts from both alleles. |

**2^nd^ round**

**Reviewer 1**

Thanks for addressing my questions and concerns. The new Figure 1 is very helpful and clearly summarizes the methods proposed in this work. The updated manuscript has been improved and easier to follow for the audience. I have no further questions or comments.

**Reviewer 2**

All reviewers' comments have been adequately addressed. I in particular appreciate the addition of the new figures. I therefore suggest an acceptance and would congratulate the authors.
